# Pulmonary delivery of docosahexaenoic acid mitigates bleomycin-induced pulmonary fibrosis

**DOI:** 10.1186/1471-2466-14-64

**Published:** 2014-04-18

**Authors:** Hongyun Zhao, Yee Chan-Li, Samuel L Collins, Yuan Zhang, Robert W Hallowell, Wayne Mitzner, Maureen R Horton

**Affiliations:** 1 Department of Medicine, Johns Hopkins University School of Medicine, 1830 East Monument Street, 5th floor, Baltimore, MD, USA; 2 Departments of Environmental Health Sciences, Division of Physiology, Johns Hopkins University Bloomberg School of Public Health, Baltimore, MD, USA; 3 Department of Respiratory Medicine, Shanghai Pulmonary Hospital, School of Medicine, Tongji University, Shanghai, China

## Abstract

**Background:**

Pulmonary fibrosis is an untreatable, fatal disease characterized by excess deposition of extracellular matrix and inflammation. Although the etiology of pulmonary fibrosis is unknown, recent studies have implicated dysregulated immune responses and wound healing. Since n-3 polyunsaturated fatty acids (n-3 PUFAs) may beneficially modulate immune responses in a variety of inflammatory disorders, we investigated the therapeutic role of docosahexaenoic acid (DHA), a single n-3 PUFA, in lung fibrosis.

**Methods:**

Intratracheal DHA or PBS was administered to mouse lungs 4 days prior to intratracheal bleomycin treatment. Body weight and survival were monitored for 21 days. Bronchoalveolar fluid (BALF) and lung inflammatory cells, cytokines, eicosanoids, histology and lung function were determined on serial days (0, 3, 7, 14, 21) after bleomycin injury.

**Results:**

Intratracheal administration of DHA mitigated bleomycin-induced lung injury. Mice pretreated with DHA had significantly less weight loss and mortality after bleomycin injury. The lungs from DHA-pretreated mice had markedly less fibrosis. DHA pretreatment also protected the mice from the functional changes associated with bleomycin injury. Bleomycin-induced cellular inflammation in BALF and lung tissue was blunted by DHA pretreatment. These advantageous effects of DHA pretreatment were associated with decreased IL-6, LTB_4_, PGE_2_ and increased IL-10.

**Conclusions:**

Our findings demonstrate that intratracheal administration of DHA, a single PUFA, protected mice from the development of bleomycin-induced pulmonary inflammation and fibrosis. These results suggest that further investigations regarding the role of n-3 polyunsaturated fatty acids in fibrotic lung injury and repair are needed.

## Background

Idiopathic Pulmonary fibrosis (IPF) is a relentless and progressive disease characterized by lung inflammation and subsequent fibrosis [1,2]. A critical barrier to treating this disease is the lack of understanding of the pathophysiology driving the fibrosis. In pulmonary fibrosis, the normally self-limiting lung inflammation that results in healing and restoration of tissue integrity becomes an unremitting inflammation characterized by persistent inflammatory cell infiltration, fibroblast recruitment and ensuing fibrosis [3,4]. Although the etiology of this persistent inflammation is unclear, it is clear that this disease is associated with the persistence of pro-inflammatory cytokines and chemokines. Specifically, TNF-α, TGF-β, and both CXC chemokines (IL-8, MIG, IP-10, I-TAC) as well as CC chemokines (MIP-1, MCP-1) [5]. Recently, inflammatory lipid eicosanoid mediators, such as prostaglandins (PGs) and leukotrienes (LTs), have also been linked to pulmonary fibrosis [6-10].

Fish oil, a complex mixture of polyunsaturated fatty acids as well as a range of other unsaturated and saturated fatty acids, has been deemed beneficial in a wide variety of human chronic ailments [11-16]. Unfortunately, due to the complex mixture of fats in the fish oil, many of the purported benefits have been recently questioned [17-26]. Thus, investigating a single, pure polyunsaturated fatty acid (PUFA) is pivotal in understanding its role in diseases. Recent studies have showed that a pure n-3 PUFA may exert beneficial effects in inflammatory ailments [27-31]. The pure n-3 PUFAs have been noted to reduce inflammatory cells, regulate key inflammatory cytokines such as IL-6 and TNF-α and suppress the production of prostaglandins (PGs) and leukotrienes (LTs) [31-35]. It is believed that the beneficial effects of n-3 PUFAs are due in part to the replacement of arachidonic acid in the membrane phospholipids of inflammatory cells with the n-3 PUFAs thus leading to a reduced capacity of immune cells to synthesize LTs and PGs [36,37]. At present, the specific effects of n-3 PUFAs on pulmonary fibrosis have not been studied. In this study, we demonstrate that DHA, one best-known n-3 PUFA, protects mice from bleomycin-induced pulmonary inflammation and fibrosis. Intratracheal DHA mitigated bleomycin induced mortality, weight loss, inflammation, histological damage and loss of lung function. Overall these findings provide insight into the disease process as well as suggest a novel therapeutic approach for pulmonary fibrosis.

## Methods

### Reagents

DHA was purchased from Sigma-Alrich (St. Louis, MO), bleomycin from Hospira (Lake Forest, IL) and antibodies from eBiosceice (San Diego, CA) and Cayman (Ann Arbor, MI).

### Animals

In this study, 8 week old female C57BL/6 mice were purchased from Jackson Laboratories (Bar Harbor, ME), housed in a pathogen-free sterile facility at the Johns Hopkins School of Public Health and allowed water and food ad libitum. All animal experiments were approved by the Johns Hopkins Animal Care and Use Committee.

### Pulmonary fibrosis model

8 week old female C57BL/6 mice were anesthetized by intraperitoneal injection of 75 mg/kg ketamine and 5 mg/kg xylazine, intubated and given 50 μl of PBS or DHA (6.25 mg/kg body weight) diluted in PBS intratacheally. 4 days after DHA or PBS treatment, mice were again anesthesized, intubated and given intratracheal bleomycin (1.5 U/kg or 2.5 U/kg) in 50 μl PBS. Body weight and survival were monitored. Mice were sacrificed for bronchoalveolar lavage, FACS, histology and lung function measurements at days 0, 3, 7, 14 and 21 after bleomycin. Day 0 represents baseline, i.e. 4 days after DHA or PBS, but prior to bleomycin.

### BALF cell count

BALF differential cell counts were carried out through cytopspin preparation (Thermo Scientific, Waltham, MA) and Diff-Quick Staining (Dade Behring; Germany).

### Flow cytometry

All flow cytometry of lung cells was performed on a Facs Caliber, BD Biosciences (San Jose, CA). FACS antibodies were purchased from eBiosceice (San Diego, CA).

### ELISA

IFN-γ, TNF-α, IL-1, IL-6, IL-10 and IL-33 were measured in BALF by ELISA according to the manufacturer’s protocol (eBioscience, San Diego, CA). Leukotriene B_4_ (LTB_4_), and prostaglandin E2 (PGE_2_) were measured with immunoassays per protocol from Cayman (Ann Arbor, MI).

### Lung function measurement

Using the forced oscillation technique, tissue elastance (H) and tissue damping (G) that reflect the severity of pulmonary fibrosis were measured [10,38]. All dynamic measurements were performed in vivo on anesthetized mice (intraperitoneal 100 mg/kg ketamine and 10 mg/kg xylazine) with an intact chest wall. After animals were anesthetized, tracheotomy and intubation was performed and the animals were ventilated by a computerized Flexivent system (SCIREQ Scientific respiratory equipment, Montreal, Quebec, Canada) with a tidal volume of 0.2 ml of 100% oxygen at a rate of 150 breaths/min. After administration of succinylcholine (2 mg/kg), a deep inspiration (DI) was given and held for 5 seconds, prior to returning to normal ventilation. One minute after the DI, measurement was performed and data were fit into constant phase model to determine G and H.

### Histology and imaging

Following assessment of lung function, the lungs were inflated at a pressure of 25 cmH_2_O with formalin, sectioned and stained with Hematoxylin-eosin (H&E) for lung morphology and Masson’s trichrome to observe collagen deposition. The sections were photographed at 20X magnification to create panorama images which were scored for fibrosis by Ashcroft method as previously described [39].

### Statistics

Data were and analyzed with the Mann–Whitney U-test running Prism software (GraphPad Inc., San Diego, CA). P values < 0.05 were considered significant. Survival curves (Kaplan-Meier plots) were compared using a log rank test.

## Results

### Intratracheal DHA enhances survival following bleomycin injury

**Figure 1:**
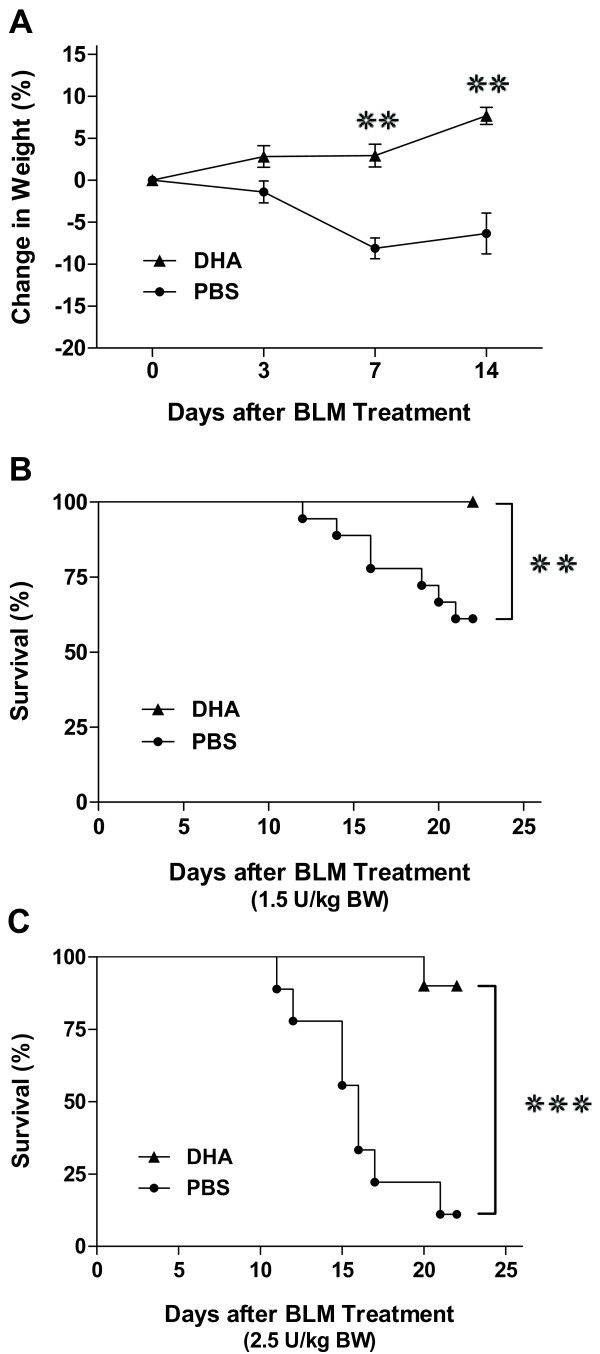
**Intratracheal DHA enhances survival following bleomycin injury.** Mice were pretreated with DHA (6.25 mg/kg BW) or PBS 4 days prior to bleomycin injury. **A**. DHA-pretreated mice did not lose any weight after bleomycin injury where as the PBS-pretreated mice lost nearly 10% of their body weight (Bleomycin dose: 1.5 U/kg BW; PBS, n = 17; DHA, n = 15). **B**. Survival curve for PBS and DHA groups (Bleomycin dose: 1.5 U/kg BW; PBS, n = 18; DHA, n = 15). **C**. Survival curve for PBS and DHA groups (Bleomycin dose: 2.5 U/kg BW; PBS, n = 9; DHA, n = 10). Values are means ± SM. **❊** P < 0.05, **❊❊** P < 0.01, **❊❊❊** P < 0.001.

We hypothesized that administration of DHA, a pure single n-3 polyunsaturated acid, would mitigate bleomycin-induced injury in mice. As pure n-3 PUFA is not stable and highly prone to oxidation, in order to effectively and reliably administer it to the lungs, we utilized direct pulmonary delivery [40]. The optimum dose and timing of DHA was determined by a pilot study and other published DHA studies [28,41]. Thus intratracheal DHA or PBS was administered to mice 4 days prior to intratracheal bleomycin. The mice displayed no adverse effects from the administration of either the DHA or PBS on day 0 (4 days after DHA or PBS but before bleomycin). After bleomycin injury, mice pretreated with DHA had significantly less weight loss (Figure 1A). In addition, there was a significant increase in survival for the DHA-pretreated groups compared to the PBS (Figure 1B-C). This survival advantage occurred with both low dose (100% vs 61%) and high dose (90% vs 11%) bleomycin (Figure 1B-C). Thus, pre-treatment of mice with intratracheal DHA prior to bleomycin significantly protected the animals’ health.

### Intratracheal DHA mitigates bleomycin-induced lung fibrosis

**Figure 2:**
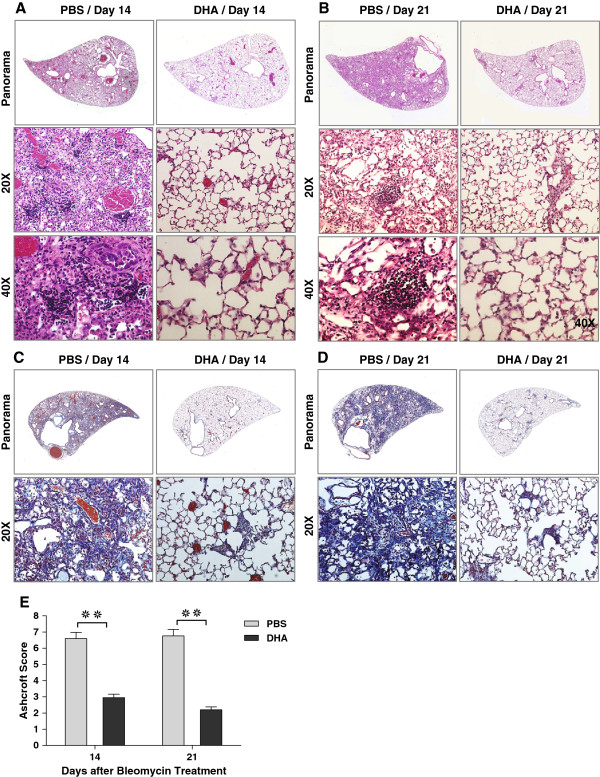
**Intratracheal DHA mitigates bleomycin-induced lung fibrosis.** Hematoxylin-eosin staining of lung tissue on days 14 **(A)** and 21 **(B)**. Masson’s trichrome staining of lung tissue on days 14 **(C)** and 21 **(D)**. **E**. Ashcroft score of tissue damage on days 14 and 21. **❊❊** P < 0.01 (For day 14: n = 5 and section = 15 for PBS group, n = 7 and section =21 for DHA group; for day 21: n = 7 and section = 21 for PBS group, n = 10 and section = 30 for DHA group).

In order to determine if the improved survival after bleomycin injury in DHA-pretreated mice was due to decreased fibrosis, histological examination and lung function measurement were performed at 14 and 21 days after bleomycin. The PBS-bleomycin-treated lungs demonstrated substantially increased fibrosis and inflammation on days 14 and 21 days (Figure 2A-D). In contrast, there was markedly less fibrosis and inflammation in DHA-pretreated mice and their lung structure showed only minimal damage (Figure 2A-D). Quantification of the histological sections after bleomycin injury demonstrated significant differences in the histology scores between DHA and control groups on days 14 and 21 after bleomycin injury (Figure 2E) [39]. There was no difference in the baseline (Day 0) histology of PBS- and DHA-pretreated mice (data not shown).

**Figure 3:**
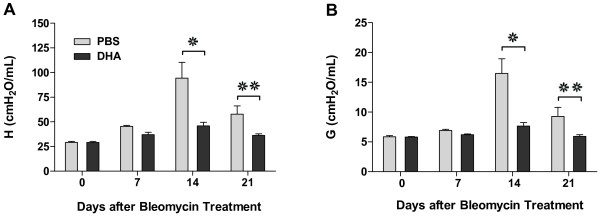
**Intratracheal DHA mitigates bleomycin-induced lung restriction.** Assessment of tissue elastance (H) **(A)** and tissue damping (G) **(B)** on days 14 and 21 as an indication of lung stiffness (restriction) post bleomycin injury. **❊** P < 0.05, **❊❊** P < 0.01 (For day 0: n = 4 for both groups; for day 7: n = 4 for both groups; for day 14: n = 5 for PBS group, n = 7 DHA group; for day 21: n = 7 for PBS group, n = 10 for DHA group).

Lung mechanics focused on the changes in lung tissue elastance (H) and tissue damping (G) from the conventional forced oscillation technique [38,42]. Tissue elastance reflects the stiffness of the lung and tissue damping reflects the energy dissipation in the lung tissues. Although there was no significant difference between the lung tissue elastance of PBS and DHA-pretreated groups on day 7 after bleomycin injury, there were marked decreases in both the tissue elastance and damping in the DHA-pretreated mice on days 14 and 21 (Figure 3). Overall, not only did DHA prevent histological fibrosis, it also prevented the decreased compliance of the lungs associated with fibrosis and inflammation.

### DHA prevents bleomycin-induced lung inflammation

**Figure 4:**
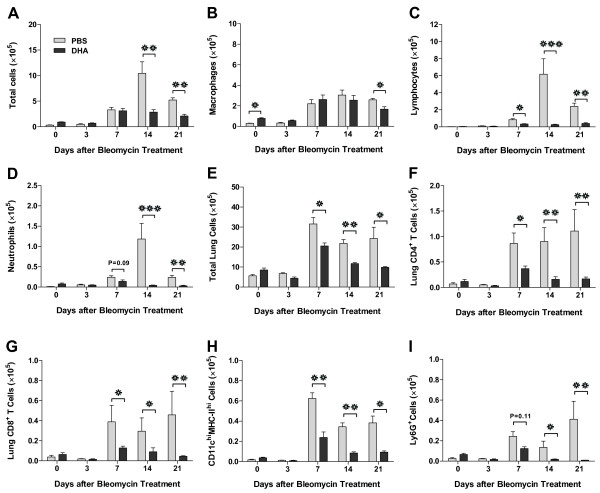
**Intratracheal DHA mitigates bleomycin-induced lung inflammation. A-D**. BALF total cell and differential cell counts in PBS-pretreated mice (grey bars) and DHA-pretreated mice (black bars) (n ≥ 5 for both groups at each time point). **E-I**. Flow cytometry analysis of total lung cells in PBS vs DHA pretreated groups. **❊** P < 0.05, **❊❊** P < 0.01, **❊❊❊** P < 0.001(for days 0 and 3: n ≥ 3 for each group; for days 7, 14 and 21: n ≥ 5 for each group).

In order to determine if the effects of DHA were due to a decreased inflammatory response to the bleomycin, we studied the bronchoalveolar fluid (BALF) and total lung from DHA-pretreated mice before and after bleomycin injury. On day 0 (4 days after DHA or PBS but before bleomycin), the total cell count was similar in the DHA- and PBS- pretreated BALF but there was a slight increase in the numbers of macrophages in the DHA group (Figure 4A-D). In response to bleomycin, both groups initially responded similarly on days 3 and 7 with a mild increase in total BALF cells (Figure 4A). However, beginning on day 14 and persisting until day 21, the PBS-pretreated mice had a marked increase in BALF total cells that was absent in the DHA-pretreated animals. Furthermore, the differential of the BALF cells differed markedly as the DHA-pretreated mice had significantly less neutrophils and lymphocytes beginning on day 7 and continuing through day 21 (Figure 4C-D).

Similar results were also noted for the cellular infiltrate in the total lung parenchyma as analyzed by flow cytometry (Figure 4E-I). Compared with the PBS-treated mice, DHA-pretreated mice revealed a blunted response in T cells (both CD4^+^ and CD8^+^) and neutrophils (Ly6G^+^) (Figure 4E-I). Interestingly, we also noted a significant decrease in lung dendritic cells (CD11C^hi^ MHC-II^hi^) after DHA exposure (Figure 4H). Thus, DHA pre-treatment blunted bleomycin-induced cellular inflammation in BALF and lung tissue most notably by inhibiting accumulations of neutrophils, T cells and dendritic cells.

### DHA inhibits bleomycin-induced inflammatory cytokines and eicosanoids

**Figure 5:**
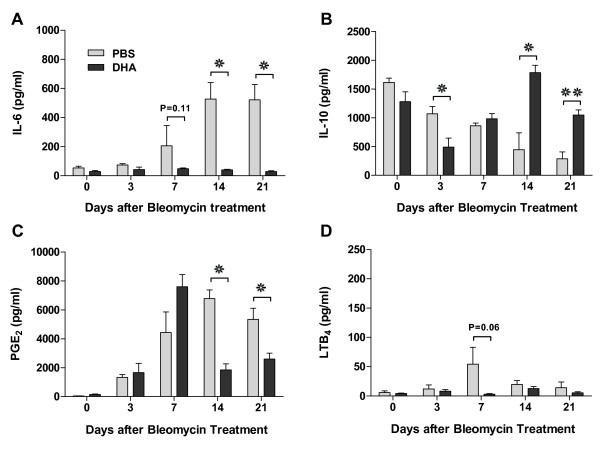
**Intratracheal DHA alters bleomycin-induced cytokines and eicosanoid mediators.** Levels of IL-6 **(A)**, IL-10 **(B)**, PGE2 **(C)** and LTB4 **(D)** in BALF from PBS vs DHA pretreated bleomycin injured mice. **❊** P < 0.05, **❊❊** P < 0.01 (n ≥ 4 for each group at each time point).

In order to determine how DHA may inhibit bleomycin–induced lung inflammation, fibrosis and mortality, we interrogated the BALF from DHA- or PBS- pretreated mice on serial days after bleomycin injury. DHA significantly impaired IL-6 expression in BALF after bleomycin (Figure 5A). In addition, there was a marked alteration in IL-10 expression. In PBS-pretreated mice, there was a continuous decrease in BALF IL-10 after bleomycin injury (Figure 5B). In contrast, in DHA-pretreated mice, although these was an initial dip in IL-10 on day 3 after injury, the IL-10 levels rebounded and remained persistently high starting on day 7 (Figure 5B). Of note, we were unable to measure significant levels of IFN-γ, IL-33, TNF-α or IL-1β in either DHA- or PBS- treated mice (data not shown). We also evaluated changes in eicosanoid mediators PGE2 and LTB4. Although PGE2 levels in both groups initially increased, DHA-pretreated animals quickly displayed decreased levels of PGE2 by day 14 after injury (Figure 5C). Interestingly, LTB4 levels remained quite low in the DHA-pretreated mice but trended to peak in the PBS-pretreated mice on day 7 after bleomycin (Figure 5D). Thus, DHA pre-treatment decreased the pro-inflammatory IL-6 and LTB_4_ by day 7 and PGE_2_ at later time points but increased the anti-inflammatory IL-10 after bleomycin injury.

## Discussion

Pulmonary fibrosis is a chronic, progressive interstitial lung disease with a poor prognosis for which there is no effective treatment [1,43]. The lack of therapy is in part due to the limited knowledge concerning the pathogenesis of the disease. However, many recent studies have implicated dysregulated immune responses and aberrant wound healing as playing key roles in the etiology of lung fibrosis. As n-3 polyunsaturated fatty acids, such as DHA (docosahexaenoic acid), have the capacity to modulate immune responses with beneficial effects, we hypothesized they may have a therapeutic role in lung fibrosis. We demonstrate the ability of intratracheal DHA to mitigate bleomycin-induced lung injury. Specifically, mice pretreated with DHA have significantly less weight loss, mortality, lung inflammation, histology damage and physiologic derangements in lung function. Mechanistically, DHA pretreatment appears to mitigate lung damage by suppressing lung inflammatory cells, decreasing IL-6, LTB_4_, PGE_2_ and increasing IL-10. Thus, our findings demonstrate that intra-tracheal administration of the n-3 polyunsaturated fatty acid, DHA, protected mice from the development of bleomycin-induced pulmonary inflammation and fibrosis. Our data suggest further investigations into the role of n-3 polyunsaturated fatty acids in the treatment of lung fibrosis.

While many studies claimed the beneficial effects of fish oil, we chose to look only at the pure n-3 PUFA. Given the contradictory results of many recent studies using fish oil, a complex mixture of polyunsaturated fatty acids as well as a range of other unsaturated and saturated fats, a study of such a complex material would have little chance to define specific protective pathways in lung fibrosis. Indeed, similar discrepancy also occurred in the studies about the effect of fish oil on bleomycin-induced pulmonary fibrosis [44,45]. Although the mechanism by which n-3 PUFAs decreased inflammation is unclear, changes in the composition of the cell membrane have been implicated. The cell membrane composition of inflammatory cells in subjects fed n-3 PUFA-rich diets demonstrate increased amount of n-3 PUFAs thus altering the fatty acid composition of cellular phospholipids [36,46,47]. Although little is known about the effects of these cellular modifications by DHA, Fan et al. have suggested that the impacts of n-3 PUFAs on membrane micro-domains may regulate intracellular signal transduction (18). Our study is unique in that we directly administered the DHA to the target organ, the lungs. We gave the DHA 4 days before the bleomycin to allow significant time for the n-3 PUFA to integrate into the membrane phospholipid and mitigate any effect due only to the intratracheal instillation itself. As previous studies have demonstrated that the integration of the DHA into the membrane is stable for at least 8 weeks, thus the impact of the DHA on membrane composition would still be maintained during the 21 days after bleomycin injury [36].

Although no animal model of lung fibrosis is perfect, the bleomycin model is a well-established, extensively studied, reproducible model of murine lung fibrosis that can be interrogated to reveal potential mechanisms underlying human fibrotic lung disorders [48-51]. DHA pretreatment significantly mitigated the fibrotic changes induced by bleomycin on examination of histology, lung function and mortality. Both panorama and high power images of lung tissue demonstrate a clear decrease in fibrosis in the DHA-treated animals. The ability of n-3 PUFAs to inhibit inflammation has been suggested in numerous diseases such as Crohn’s Desease and rheumatoid arthritis [11,13]. Indeed, we demonstrate that DHA also prevented the bleomycin-induced inflammatory cells in both BALF and lung tissue, in particular there was a marked decrease in lymphocytes and neutrophils in the lungs of DHA-pretreated animals.

We also demonstrate a change in the inflammatory cytokine profile in DHA-pretreated bleomycin injured mice. While there is a marked rise in the IL-6 level of PBS-bleomycin-treated mice, the DHA-pretreated mice have no increase in IL-6 levels. This is in keeping with recent studies displaying that DHA inhibited macrophage IL-6 production [31,34]. Although the role of IL-6 in pulmonary fibrosis is only emerging, Saito et al. have reported less severe bleomycin-induced lung injury in IL-6-deficient mice [52]. Additionally IL-6 has been implicated in mediating the phenotypic conversion of fibroblast to myofibroblast via up-regulation of α-SMA [53]. As lung myofibroblasts appear to play a role in lung fibrosis, DHA may be mitigating the development of fibrosis by inhibiting bleomycin induced IL-6 [54-56]. Lastly, blocking of IL-6 receptor has also been shown to alleviate bleomycin-induced skin fibrosis [57].

In addition, we also demonstrate changes in IL-10 expression in DHA-treated animals. In the PBS-bleomcyin-treated controls, the IL-10 levels progressively decrease as the inflammation and fibrosis develop and are quite low by day 21. However, in the DHA-bleomcyin-treated animals, although the IL-10 levels dip on day 3, they begin to increase thereafter and are back to or above baseline levels by days 14 and 21. The exact role of IL-10 in pulmonary fibrosis is unclear. While gene delivery of IL-10 has been showed to attenuate pulmonary fibrosis other investigators have found it only inhibited inflammation, but not fibrosis [58,59]. Regardless, in our study we see both preserved IL-10 levels at late time points correlating with decreased inflammation and fibrosis in the DHA-pretreated animals.

Leukotrienes (LTs) and prostaglandin (PGs) belong to a family of bioactive lipids, called eicosanoids that are produced from arachidonic acid by 5-lipoxygenase and cyclo-oxygenase [6]. Eicosanoid mediators have been suggested to be involved in a host of inflammatory diseases, and their potential role in pulmonary fibrosis has been increasingly recognized [6,10,60]. Incorporation of n-3 PUFAs, in place of arachdonic acid into cell membranes, results in decreased production of eicosanoid mediators as n-3 PUFAs are poor substrates for 5-lipoxygenase and cyclo-oxygenase [36,37]. Several human studies reveal that dietary n-3 PUFA supplementation significantly decreased PGE_2_ and LTs production by human immune cells [36,61]. Indeed, our data demonstrating decreased PGE_2_ at later time points after bleomycin injury in the DHA-treated animals is consistent with studies demonstrating that DHA inhibit PGE_2_ synthesis [27,32,33,35].

Currently, the role of PGE_2_ in pulmonary fibrosis is unclear. Both human and animal studies alike have demonstrated seemingly contradictory data concerning the beneficial or detrimental effects of prostaglandins in lung fibrosis [9,10,62-65]. While several investigators have demonstrated that exogenously increasing PGE_2_ levels protected mice from bleomycin-induced lung injury, others have suggested that PGI_2_, not PGE_2_, is the protective prostaglandin [9,10,63]. Our finding of lower levels of PGE_2_*in* DHA-treated mice on days 14 and 21, as the acute inflammation recedes and the fibrosis begins, may indicate that PGE_2_ may be pathogenic in the fibrotic process. Our study differs from other studies purporting the benefits of PGE_2_ in mitigating lung fibrosis in many ways. We are administering the DHA, not PGE_2_, thus affecting upstream pathways not just PGE_2_ levels. We measure PGE_2_ in BALF at many time points during the acute inflammation and subsequent fibrosis. Other studies either do not measure PGE_2_ content or only at early time points where we too see no difference [9,63,66]. Finally we are administering the DHA directly to the lungs thus avoiding any of the off target anti-inflammatory effects of administering PGE_2_ via subcutaneous pumps [9,63] Although Ivanova et al. did administer PGE_2_ by aerosolized liposomes, no BALF levels were reported making it difficult to compare directly to our data [66].

Furthermore, in a model of bleomycin-induced skin fibrosis, investigators showed mice lacking the PGE_2_ (mPGES-1 null mice) were resistant to bleomycin-induced skin fibrosis [67]. In our study, we see similar levels of PGE_2_ in the first 7 days after bleomycin injury but these levels markedly decrease in the DHA-treated mice at late time points. Although the decreased PGE_2_ levels may be mirroring the decreased inflammation and fibrosis at later time points, the relative lack of it may also prevent fibrosis as suggested in the studies showing less bleomycin-induced skin fibrosis in PGE_2_-deficient mice. Interestingly, our data also suggested there is less LTB4 in the DHA-pretreated mice. This is in keeping with the literature that demonstrated decreased bleomcyin-induced fibrosis in mice deficient in leukotrienes or in those mice where LTB_4_ receptor is blocked [7,60]. Thus, as DHA is known to modify cell membrane composition and thus eicosanoids metabolism, in our model DHA pre-treatment may be mitigating the bleomycin-induced lung inflammation and fibrosis by altering the eicosanoids, LTB_4_ and PGE_2_.

## Conclusion

In conclusion, our findings demonstrate that intra-tracheal administration of the n-3 polyunsaturated fatty acid, DHA, protected mice from the development of bleomycin-induced pulmonary inflammation and fibrosis. Our data suggest further investigations into the role of n-3 polyunsaturated fatty acids in the treatment of lung fibrosis.

## Competing interests

The authors declare that they have no competing interests.

## Authors’ contributions

HZ and MRH conceived and designed these studies; HZ and YCL performed experiments, HZ, YCL and MRH analyzed data; MRH, HZ, YCL, SLC, YZ, RWH and WM interpreted results of experiments; HZ and MRH drafted manuscript; MRH, HZ and WM edited and revised manuscript; All authors read and approved the final version of manuscript.

## Pre-publication history

The pre-publication history for this paper can be accessed here:

http://www.biomedcentral.com/1471-2466/14/64/prepub
